# Intraoperative Diagnosis of Stanford Type A Dissection by Transesophageal Echocardiogram in a Patient Presenting for Renal Transplantation

**DOI:** 10.1155/2011/263561

**Published:** 2012-01-11

**Authors:** William R. Hand, John S. Ikonomidis, Charles F. Bratton, Thomas M. Burch, Matthew D. McEvoy

**Affiliations:** ^1^Department of Anesthesiology and Perioperative Medicine, Medical University of South Carolina, Charleston, SC 29425, USA; ^2^Department of Surgery, Medical University of South Carolina, Charleston, SC 29425, USA; ^3^Department of Anesthesiology, Wake Forest University, Winston-Salem, NC 27157-1009, USA

## Abstract

A 48-year-old patient with hypertensive end-stage renal disease presented for cadaveric renal transplantation. On physical exam, a previously undocumented diastolic murmur was heard loudest at the left lower sternal border. The patient had a history of pericardial effusions and reported “a feeling of chest fullness” when lying flat. As such, a transesophageal echocardiogram (TEE) was performed after induction of anesthesia to evaluate the pericardial space and possibly determine the etiology and severity of the new murmur. The TEE revealed a Stanford Type A aortic dissection. The renal transplant was cancelled (organ reassigned within region), and the patient underwent an urgent ascending and proximal hemiarch aortic replacement. This case demonstrates the importance of a thorough physical exam and highlights the utility of TEE for noncardiac surgical cases.

## 1. Case

A 48-year-old African-American male with a past medical history significant for end-stage renal disease (ESRD) secondary to poorly controlled hypertension presented for cadaveric renal transplantation. The patient underwent dialysis three times per week and had been on the transplant list for approximately one year. He denied chest pain, dyspnea on exertion, or syncope. He reported 3-pillow orthopnea, lower extremity edema, and a ‘‘feeling of fullness in my chest if I lay flat.” Vital signs in the preoperative area were heart rate 78, blood pressure 155/74, respiratory rate 20, and oxygen saturation 98% on room air. His pain score was 0/10. Auscultation of the chest revealed clear breath sounds (louder on right), a pericardial friction rub heard best at base, distant first and second heart sounds, and a II/VI diastolic murmur heard best at the lower left sternal boarder. Chest X-ray revealed an enlarged pericardial silhouette, unchanged from prior films. A transthoracic echocardiogram (TTE) from two years earlier revealed a chronic circumferential pericardial effusion that was not hemodynamically significant at that time. No valvular abnormalities were noted on the TTE.

Concerns of a possible pericardial effusion and aortic insufficiency (AI) were discussed with the patient and the transplant surgeon. An intraoperative transesophageal echocardiogram (TEE) was planned in order to evaluate the suspected effusion and aortic valvular insufficiency (AI). This approach was taken in order to recognize any hemodynamic impairment from the pericardial effusion or possible AI after the induction of anesthesia and institution of positive-pressure ventilation. If the effusion compromised ventricular filling and cardiac output, cardiology would be available to perform a needle pericardiocentesis and then the renal transplant would proceed. If there were no hemodynamic sequelae evident by vital signs or TEE, the renal transplant would proceed with a plan to evaluate the effusion postoperatively, knowing that it would most likely resolve over the ensuing weeks to months with the discontinuation of dialysis and proper function of the transplanted kidney. A preoperative TTE was not obtained in order to minimize the ischemic time of the donor kidney and because the patient did not report symptoms suggesting severe AI.

**Figure 1:**
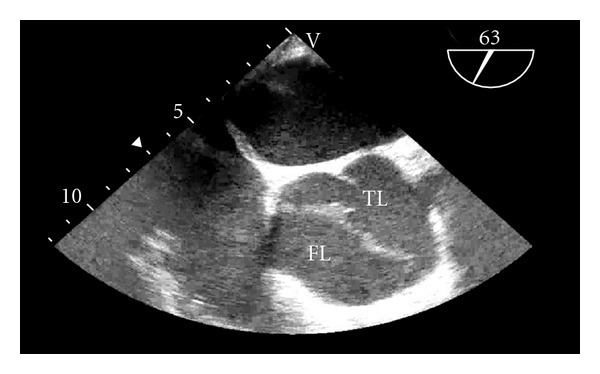
TEE image of the proximal ascending aorta (approximately at level of sinotubular junction) in mid-esophageal short axis view. The true lumen (TL) can be seen with the right, left, and noncoronary cusps. The false lumen (FL) is clearly obvious and occupies approximately forty percent of the aortic luminal area. This pathology caused severe aortic insufficiency which was demonstrable with color flow doppler (not shown).

**Figure 2:**
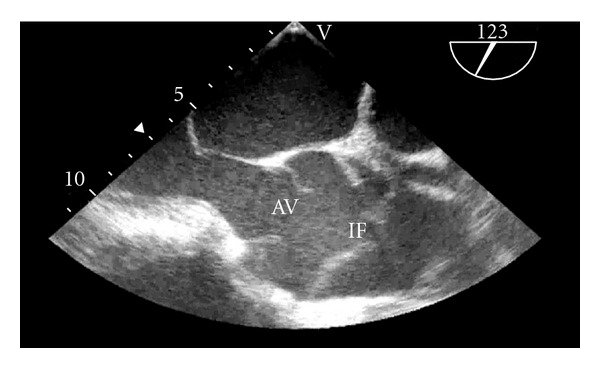
TEE image of the aortic valve (AV) and root in mid-esophageal long axis view. The dissection is visible just distal to the sinotubular junction. Notice that the intimal flap (IF) traverses the width of the proximal aorta.

After placement of an awake arterial line and a stable induction of anesthesia, the TEE probe was inserted and a Stanford Type A dissection was discovered (see Figures 1 and 2). The transplant surgeons notified that the renal transplant would not be performed. The organ was able to be transplanted into another patient in the region. The Cardiothoracic Surgery service was immediately consulted, resulting in a decision to proceed with an ascending and proximal hemiarch aortic replacement. Emergence from anesthesia was attempted in order to discuss this development with the patient. However, the patient became hypertensive and tachycardic during this attempt, even with use of extensive beta-blockade and other antihypertensive treatment. Thus, emergency consent was obtained from the patient's wife and the patient underwent immediate ascending and proximal hemiarch aortic replacement with deep hypothermic circulatory arrest and cardiopulmonary bypass. The patient tolerated this procedure well and was discharged on postoperative day six.

Of note, after the surgical repair, and upon further questioning, the patient reported that he did have “a tearing feeling in his back between his shoulder blades.” He reported that “it started about five days ago when I was helping my wife carry in some groceries.” When asked why he did not report this during the preoperative interview, he stated, “I was concerned that I wouldn't be able to get my kidney transplant.”

## 2. Discussion

This case illustrates the importance of obtaining an accurate and comprehensive history and physical exam. In the current age of information and technology-driven healthcare, the emphasis on physical exam acumen has diminished [1]. Transplant recipients often have multiple comorbidities with abnormal physical findings. Incidence of calcification of multiple intracardiac valves and/or the aorta is known to occur in as much as 47% of this population [2]. These pathologic processes often present with physical findings. Comparing the current physical exam to those noted in the medical history requires vigilance, especially in the time-sensitive setting of an ischemic donor organ.

Diastolic murmurs are heard between the second heart sound of one cardiac cycle and the first heart sound of the next. All diastolic murmurs are pathologic; thus, auscultation of such a murmur should always be investigated. The common diastolic murmurs in adults include those found with aortic valvular regurgitation and mitral or tricuspid stenosis, along with that heard with an atrial myxoma. As detailed above, the diastolic murmur heard during the preoperative exam, in addition to the history of a pericardial effusion, led the providers to a heightened level of concern about the cardiovascular stability of the patient about to undergo kidney transplantation. We chose not to obtain a TTE preoperatively in order to minimize the ischemic time of the donor kidney. Had the TEE probe not been inserted, the patient may have suffered considerable morbidity and perhaps mortality given the severity of the undiagnosed aortic dissection [3].

Aortic dissection is defined as a separation of the layers of the aortic wall extending proximally and distally. In 90% of patients, the separation results from an intimal tear. Dissections are classified by the location of the tear and the degree of aortic. The intimal tear usually occurs in the ascending aorta near the sinotubular junction (60–70%, Stanford A) or in the descending aorta just distal to the left subclavian at the site of the ligamentum arteriosum (16–30%, Stanford B) [3, 4]. Differentiation between type A and B dissections is important, because type A dissections have a lower mortality with surgical versus medical therapy, while type B dissections have equivalent outcomes following surgical or medical management [5]. Untreated acute type A dissections have a two-day mortality of up to 50% and a three-month mortality approaching 90%, thus requiring urgent or emergent surgical therapy [3]. Death following dissection most commonly results from rupture of the false lumen into the pleural or pericardial space. Interruption of vital organ perfusion also contributes to morbidity and mortality [4].

Risk factors for aortic dissection include hypertension, age greater than sixty, male gender, bicuspid aortic valve, and connective tissue diseases, most of which are present and accelerated in patients with ESRD [3]. TEE allows rapid intraoperative diagnostic confirmation of the presence or absence of a dissection, as well as giving real-time information about ventricular function/filling and valvular pathology. Although computed tomography and magnetic resonance imaging continue to improve which proved vital in this situation, TEE has the advantage of portable, rapid bedside assessment of the critically ill perioperative patient. Echocardiographic visualization of aortic dissections has a sensitivity of 97–99% and a specificity of 70–100% [6, 7].

## 3. Conclusion

While the availability of TEE is becoming more widespread and its utility in noncardiac surgery continues to gain support, it should not supplant the application of diagnostic reasoning after vital information has been gathered in the history and physical exam [8]. In this case, TEE allowed for a rapid, reliable bedside assessment of a previously undiagnosed critical condition in a renal transplant patient. Unfortunately, the patient was exposed to unnecessary risk. A new diastolic murmur should have been evaluated with TTE prior to proceeding with this case. This would have led to proceeding with an urgent aortic arch repair and the organ would have been diverted to another patient and possibly diminished the ischemic time for the renal allograft. As the initial preoperative evaluation is often remote from presentation for transplant, this case highlights the ongoing need for thorough preoperative work-up of patients with ESRD and the constant vigilance needed in taking care of patients who are at high risk for other comorbidities.

##  Disclosure

There were no financial incentives, real or potential, for any of the authors.
